# Systems for the Functional Evaluation of Human Heart Tissues Derived from Pluripotent Stem Cells

**DOI:** 10.1093/stmcls/sxac022

**Published:** 2022-03-18

**Authors:** Kozue Murata, Hidetoshi Masumoto

**Affiliations:** 1 Clinical Translational Research Program, RIKEN Center for Biosystems Dynamics Research, Kobe, Japan; 2 Department of Cardiovascular Surgery, Graduate School of Medicine, Kyoto University, Kyoto, Japan; 3 Institute for Advancement of Clinical and Translational Science, Kyoto University Hospital, Kyoto, Japan

**Keywords:** organ-on-a-chip, pluripotent stem cell, cardiomyocyte, cardiotoxicity

## Abstract

Human pluripotent stem cells (hPSCs) are expected to be a promising cell source in regenerative medicine and drug discovery for the treatment of various intractable diseases. An approach for creating a 3-dimensional (3D) structure from hPSCs that mimics human cardiac tissue functions has made it theoretically possible to conduct drug discovery and cardiotoxicity tests by assessing pharmacological responses in human cardiac tissues by a screening system using a compound library. The myocardium functions as a tissue composed of organized vascular networks, supporting stromal cells and cardiac muscle cells. Considering this, the reconstruction of tissue structure by various cells of cardiovascular lineages, such as vascular cells and cardiac muscle cells, is desirable for the ideal conformation of hPSC-derived cardiac tissues. Heart-on-a-chip, an organ-on-a-chip system to evaluate the physiological pump function of 3D cardiac tissues might hold promise in medical researchs such as drug discovery and regenerative medicine. Here, we review various modalities to evaluate the function of human stem cell-derived cardiac tissues and introduce heart-on-a-chip systems that can recapitulate physiological parameters of hPSC-derived cardiac tissues.

## Graphical Abstract

**Graphical Abstract:**
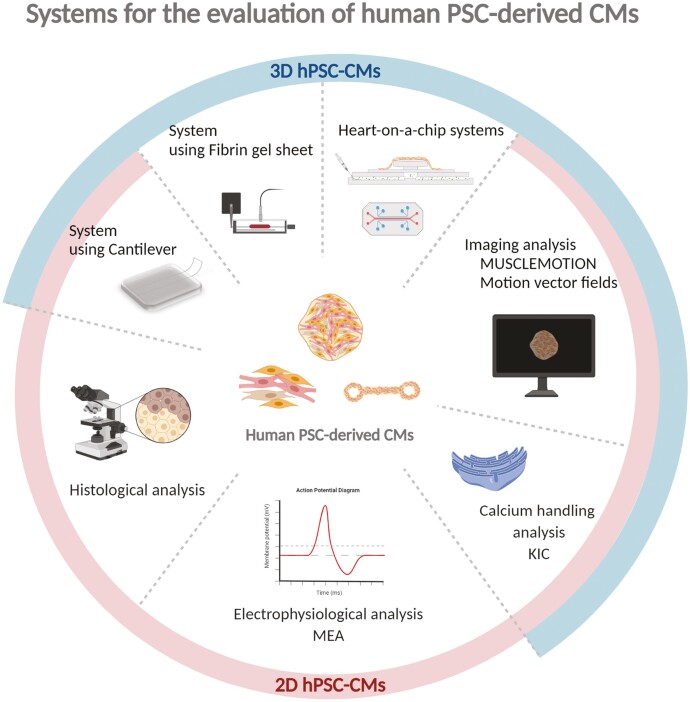

**Significance Statement**

Human pluripotent stem cells (hPSCs) are a promising cell source in regenerative medicine and drug discovery. This paper summarizes various methods for evaluating the function of human stem cell-derived cardiomyocyte tissue. The authors explore the characteristics and problems of functional assessment of stem cell-derived cardiomyocytes in two and three-dimensional culture and provide background for the advancement of research using stem cell-derived cardiac tissue.

## Introduction

Despite recent advances in its treatment, cardiovascular disease is the leading cause of death and medical expenses worldwide.^1^ Heart transplantation and ventricular assist devices for circulatory support are effective in the treatment of severe heart disease. However, they have limitations due to the shortage of donors for heart transplantation as well as the risk of complications such as thromboembolism, bleeding, and infection associated with the long-term use of ventricular assist devices. Therefore, developing a new alternative treatment for cardiovascular disease is highly anticipated.^2^ Beta-adrenergic blockers and angiotensin receptor blockers are the most commonly used treatment for heart failure, and large-cohort clinical trials have demonstrated their efficacy in preventing mortality, as well as major adverse cardiovascular and cerebrovascular events.^3,4^ However, the effect of these drugs in advanced stages of cardiovascular disease is still limited; hence, the development of more effective drugs to improve the clinical outcome of severe heart disease is desired.

There are 2 main categories of drug discovery research: one aims to search for compounds that are effective against the target disease (drug efficacy search), while the other aims to ensure the safety of the compounds that are developed (safety evaluation). To develop useful drugs, it is important to comprehensively evaluate their efficacy, safety, pharmacokinetics, and physical properties from the early development stage. For this reason, in vitro cell assays are becoming increasingly important as evaluation methods to narrow down compounds during a drug efficacy search. However, the safety evaluation of drugs currently relies on animal models for non-clinical studies. Some species barriers cannot be overcome in animal experiments, and in some cases, such as in polypharmacy, problems occur only after the drug has been administered. Therefore, the development of human-type in vitro cell assays with high fidelity is required for effective safety evaluation.

Most cardiomyocytes (CMs) in the heart are mature cells that have undergone terminal differentiation. Hence, the use of primary human cell lines as a human-type in vitro cell assay system to develop therapeutic drugs for cardiac diseases has been limited due to the difficulty of isolating adult human CMs and their expansion in culture. In recent years, there has been increasing attention on the use of pluripotent stem cells to overcome the limitations of heart failure treatment. Pluripotent stem cells (PSCs) are expected to be a viable cell source in regenerative medicine and for the study of various diseases because of their theoretically unlimited proliferation capability and the potential to differentiate into various types of somatic cells. In cardiac regenerative medicine, various methods have been reported to differentiate CMs and other cardiovascular cell types from embryonic stem cells (ESCs) collected from the inner cell mass of the blastocyst and induced pluripotent stem cells (iPSCs) established from somatic cells. The differentiation of PSCs into CMs can be regulated through varying culture conditions such as monolayers or embryoid bodies in different growth media with or without serum.^5–8^ Human PSC-derived cardiomyocytes (hPSC-CMs) are considered a promising cell source for replenishing stem cells and derivatives of cardiovascular cell lineages to restore the structure and function of damaged heart tissue.^9–13^

In drug discovery research, the use of CMs derived from human iPSCs has facilitated the use of human cells with higher fidelity. According to CIOMS pharmacogenetics, 34 newly developed drugs were withdrawn from the market for safety reasons from 1990 to 2004.^14^ These drugs were withdrawn because they caused cardiac complications, such as long QT syndrome, a potentially arrhythmogenic condition caused by cisapride, a drug for reflux esophagitis. Due to the differences in drug responsiveness because of differences in the structure and function of the heart between animals and humans, the failure of animal studies to accurately predict human cardiotoxicity was also considered to be a major reason for drug withdrawal.^15^ Thus, the evaluation of cardiotoxicity is a critical factor in the development of new therapeutic drugs.^16,17^ An attempt to evaluate cardiotoxicity using iPSC-derived cardiomyocytes (iPSC-CMs) has been conducted by an international consortium called the comprehensive in vitro proarrhythmia assay (CiPA) mainly organized by the U.S. Food and Drug Administration (FDA),^18,19^ which was expected to reduce the use of animal experiments and encourage the conduct of clinical trials that can faithfully evaluate the possible reactions of drugs in the human heart. However, there are still challenges that need to be addressed to ensure that these cell sources can become acceptable alternatives to conventional clinical trials.

The remaining concerns in the application of hPSC-CMs in drug discovery research that focus on the evaluation of cardiotoxicity are (1) the immaturity of hPSC-CMs itself, (2) the inability of hPSC-CMs to reproduce native cardiac tissue behavior caused by the lack of cell–cell communication between multiple cell types, and (3) the insufficient establishment of systems that can evaluate physiological cardiac tissue function, such as pulsatile force and pump function. Current several hPSC-based analyses with high-throughput methods can provide surrogate metrics for cardiac contractility, electrophysiology, and force generation,^20–22^ however, they are insufficient because they do not directly detect pulsation forces. The development of a system that can capture changes other than contractility or response to electrical stimulation is an important requirement to not only evaluate cardiotoxicity in drug discovery research but also the functional and structural fidelity of heart tissues derived from hPSCs for regenerative medicine.

In this review, we introduce reported methods to differentiate CMs from hPSCs, functional assessment of hPSC-CMs aiming for the evaluation of cardiac toxicity of drugs, and conventional methods for evaluating the function of cardiac tissue other than that of a single hPSC-CMs. We compared the features and limitations of methods for assessing of hPSC-CMs in 2-dimensional (2D), 3-dimensional (3D), structure and heart-on-a-chip systems which were developed to address the concerns mentioned earlier by evaluating the physiological functions of hPSC-derived 3D cardiac tissue.

## Methods of the Differentiation of CMs from Human PSCs

To drive the differentiation of hPSCs from pluripotent state to a more specific cardiac fate, it is crucial to expose hPSCs to a variety of growth factors with specific timing and dose recapitulating the development of the heart. Four signaling pathways, BMP,^23–25^ TGFB/activin/Nodal,^23,26,27^ Wnt,^7,26,28^ and FGF,^24,29,30^ are reported to play a pivotal role in regulating mesoderm cardiac specification during embryonic development, and it is necessary to carefully optimize the timing of their regulation. Currently, 2 fundamental platforms are widely used for cardiac differentiation of hPSCs, namely, embryoid body (EB) formation and monolayer culture of hPSCs. Suspension EB and forced aggregation methods, which regulate activin/nodal, BMP, and Wnt pathways, generally boast cardiomyocyte induction efficiencies of over 70% but are challenged by the inefficiency of the EBs generation process.^7,23,24,26,27^ The monolayer culture method is characterized by a high-density culture of hPSCs, induction of mesoendoderm with activin A which surrogates nodal, and the treatment with BMP which reported to yield approximately 30% of CMs induction efficiency.^25^ A modification of this method has also been reported in which Matrigel is overlaid, followed by FGF2 treatment and the administration of DKK1, a canonical Wnt antagonist at mesoderm stage which improved the efficiency of CMs induction to approximately 70%.^29^ For both EB formation and monolayer culture, the use of Wnt signaling inhibitors IWR-1 or IWP-4 at mesoderm stage has been reported to improve the induction efficiency of CMs.^26,28^

## The Application of Human PSC-Derived CMs for Cardiotoxicity Evaluation

**Figure 1.**
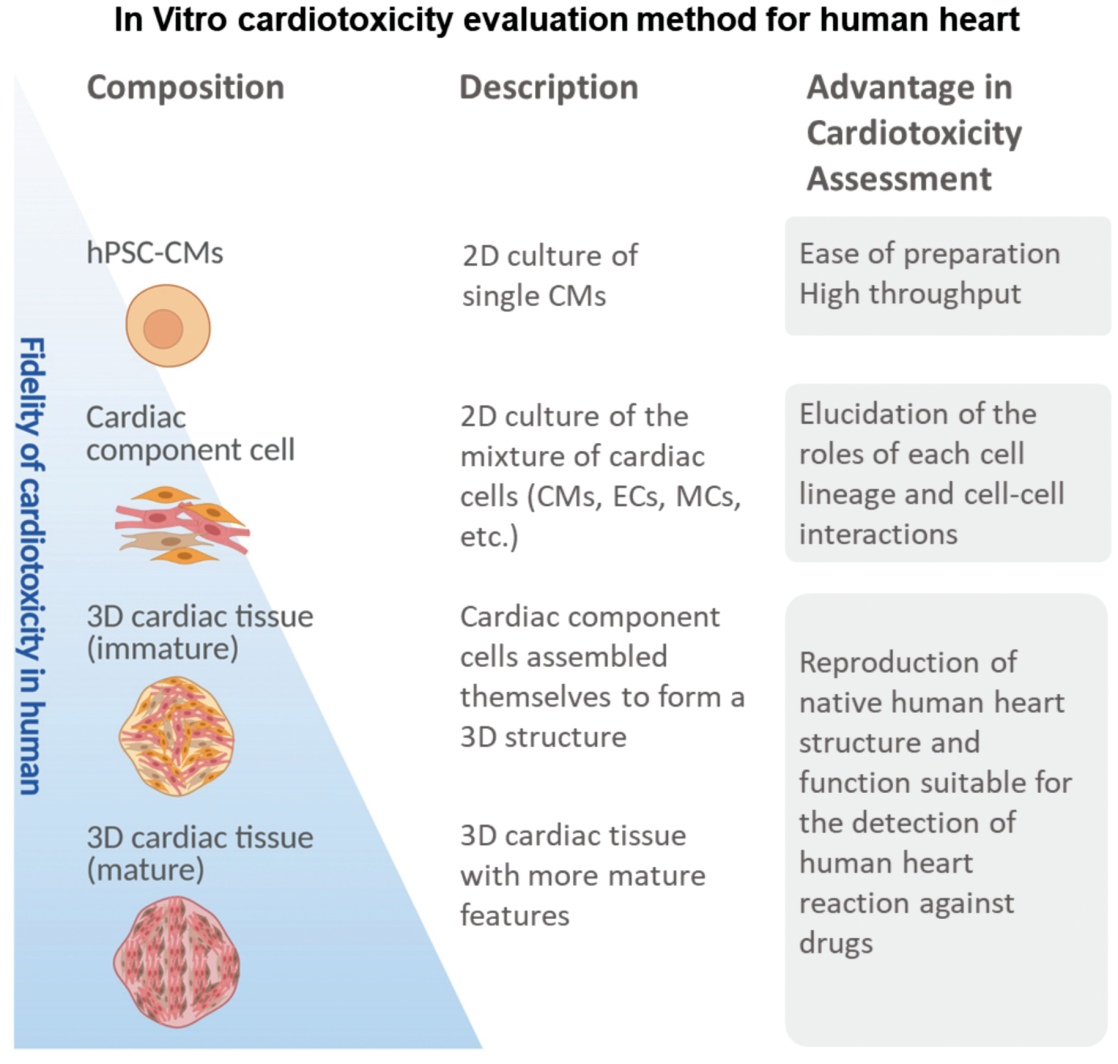
In vitro evaluation methods for cardiotoxicity in the human heart. Abbreviations: hPSCs, human pluripotent stem cells; hPSC-CMs, cardiomyocytes induced from hPSCs; CMs, cardiomyocytes; ECs, vascular endothelial cells; MCs, vascular mural cells; 2D, 2-dimensional; 3D, three-dimensional.

The hPSC-CMs induced by various methods as shown in the previous chapter is expected to promote the drug discovery. The development process of a new drug is largely divided into 2 categories, namely, non-clinical studies to evaluate drug efficacy, toxicity, and safety using animals and cells, and clinical studies to determine the efficacy of the drug by administering it to humans. In the non-clinical studies, it is required to ensure the safety of various organs. In particular, cardiac toxicity accounts for a large percentage of the reasons for withdrawal from the market, so the Safety Pharmacology Study (S7B) of the ICH (International Conference on Harmonization of Technical Requirements for Pharmaceuticals for Human Use), which is non-clinical study guidelines, requires detailed studies of the effects on cardiovascular functions.^31^ Cardiotoxicity, as defined by S7B, includes the inhibition of delayed rectifying potassium current (IKr) encoded by the human ether-a-go-go related gene (hERG) and mediated by voltage-gated potassium ion channels, resulting in prolongation of the QT interval and the appearance of the lethal arrhythmia known as Torsades de Pointes (TdP).^32^ Accurate prediction of the risk of TdP induction in drug candidates in the early drug discovery process is important to increase the probability of success in drug development. However, the hERG test, which blocked a single ion channel in non–human mammalian cells, reported that not all compounds showing strong hERG channel inhibition cause lethal arrythmias in humans.^33^ In this context, there is a need to improve the prediction technology of human TdP risk by introducing new scientific techniques. The use of hPSC-CMs is expected to improve the fidelity of human TdP-induced risk prediction because it can reproduce human electrophysiological characteristics in vitro. The comprehensive in vitro proarrhythmia assay (CiPA), an international consortium organized by the US Food and Drug Administration (FDA), aims to establish an integrated proarrhythmic risk prediction method that includes hPSC-CMs. In Japan, the Japan iPS Cardiac Safety Assessment (JiCSA) was organized and reported the usefulness of hPSC-CMs, which combines multiple ion channels expressed in the human heart, for predicting the risk of serious fatal arrhythmias.^18,19,34^ In the current international discussions on the revision of the ICH S7B guidelines, there are strong indications for the inclusion of test methods utilizing human iPS cell technology in the S7B guidelines. On the other hand, there is a remaining limitation that the evaluation methods used in these reports can only detect phenomena occurring in a single cell. However, the evaluation methods used in these guidelines can only detect phenomena occurring in a single cell. As shown in Fig. 1, single-cell cardiotoxicity assessment confers high-throughput assays, but it does not reflect cell–cell interactions. 2D cultures of cardiac cell mixtures can predict cell–cell interactions but do not accurately reflect in vivo dynamics of multiple cell interactions in cardiac tissues (Fig. 1).

The immaturity of the hPSC-CMs itself remains a limitation for its application in drug discovery research using hPSC-CMs. hPSC-CMs are reported to exhibit typical fetal-like characteristics such as small cell size, immature myofibrillar arrangement, absence of T tubules, depolarization of resting membrane potential, decreased ion channel expression, decreased upstroke velocity, fetal-like mitochondria, and glucose-dependent metabolism.^35,36^ Various attempts have been made to drive the maturation of hPSC-CMs. Physical stimulation, electrical stimulation, or the administration of additives such as fatty acids have been shown to increase the sarcomeric alignment, calcium handling, contractile force, conduction velocity, and mitochondrial content of hPSC-CMs^37–40^ (Fig. 1). It is expected that the maturation of hPSC-CMs would confer pharmacological reactivity similar to that of native heart tissue and consequently improve the accuracy of cardiotoxicity assessment.

## Evaluation Systems for the Function of 2D-Cultured Human PSC-Derived CMs

Most cardiotoxicity evaluation systems introduced so far evaluate hPSC-CMs at a single cell or 2D structural level (Table 1). For example, multielectrode array assays (MEAs)^51^ and cardiac action potentials (CAPs)^52–54^ are commonly used to monitor changes in field potentials as electrophysiological assessments. Recently, a combination of multiple parameters has been used to assess cardiotoxicity, including the assessment of beating rhythm and rate via impedance measurements such as through the xCelligence RTCA cardio system^55^ and the assessment of cellular morphological changes and viability using probes for subcellular indicators such as mitochondrial integrity and calcium homeostasis.^56^ The contraction and relaxation of CMs are determined through the change in their intracellular calcium ion concentration and the rate at which calcium ions bind to and dissociate from target proteins. Therefore, assessment of calcium handling in CMs with the green fluorescent protein (GFP)-calmodulin fusion protein (GCaMP) and rhodamine -or fluo-4 based calcium indicators is also important in the evaluation of cardiotoxicity in 2D cultures.^42,43^ The kinetic image cytometer (KIC ), developed by Cerignoli et al. is a high-throughput evaluation method that can record and analyze transient intracellular calcium concentrations in individual cells from hundreds of cells per well and has been demonstrated to be useful in the screening of new drug targets.^20,21,57^ Other evaluation systems for hPSC-CMs in 2D cultures, such as the assessment of contractility using cantilevers,^58^ a system that can evaluate the translation of sarcomere shortening to mechanical output in hPSC-CMs,^41^ and changes in the metabolic activity of CMs monolayers^59^ have been reported. These evaluation systems for 2D-cultured CMs are easy to prepare and are expected to have higher throughput compared to currently used methods. However, these evaluation systems for 2D-cultured CMs are limited to planar analysis and are often used for single cells. These systems reduce the noise during evaluation but do not allow for the evaluation of the effects on the surrounding cells, such as the paracrine effect. Moreover, 2D-cultured CMs are immature in terms of function, structure, and gene expression, and their properties are closer to fetal CMs than human adult CMs^60–63^ which might be an obstacle for the precise evaluation of the actual behavior of cardiac tissue derived from these cells.

**Table 1.** Evaluation systems for cardiotoxicity with PSC-CMs

Evaluation systems	Composition	Evaluative category	Description	Advantages	Disadvantages	Reference
Multielectrode array assays (MEA)	Single PSC-CMs 2D PSC-CMs	Electrophysiology	Capturing changes in the extracellular electric field potential of cardiomyocytes non-invasively.	Ability to assess drug effects on repolarization, depolarization, conduction, propagation. Long-term evaluation High throughput.	Lacks influence of other cardiac cell types and 3D environment of native tissue. Immature phenotype.	Clements, 2015^14^
Cantilevers	2D PSC-CMs	Contractility	Evaluation of contractility of cells spread on a thin PDMS cantilever by image analysis of cantilever shape change.	Detectable under a standard microscope.	Requires special culture devices. Not detecting the direct contractile force. Low throughput	Agarwala, 2013^58^
Analysis of the sarcomere shape and substrate stiffness	Single PSC-CMs 2D PSC-CMs	Contractility	Evaluation of contractility by integrating cell shape and substrate stiffness with other parameters.	Not requires special equipment.	Not detecting the direct contractile force. Lacks influence of other cardiac cell types and 3D environment of native tissue.	Ribeiro, 2015^41^
Calcium indicators The Kinetic Image Cytometer (KIC)	Single PSC-CMs 2D PSC-CMs 3D cardiac tissue	Contractility	Records intracellular fluorescence Ca^2+^indicators and analyzes intracellular calcium concentration.	High throughput. Not requires special equipment.	Not detecting the direct contractile force. Not fully reflect physical environment of the heart.	Cerignoli, 2012^20^ Sirenko, 2013^42^ Lewis, 2015^43^
MUSCLEMOTION	Single PSC-CMs 2D PSC-CMs 3D cardiac tissue	Contractility	Determines dynamic changes in pixel intensity between image frames and expresses the output as a relative measure of movement during muscle contraction and relaxation.	Capable of analyzing image data independent of the culture platform. Not requires special equipment. High throughput	Not detecting the direct contractile force.	Sala, 2018^44^
Analysis of motion vector fields	Single PSC-CMs 2D PSC-CMs 3D cardiac tissue	Contractility	Provides information not only on beat rate but also yields vector fields to quantify the spatial distribution of beating of tissue constructs.	Capable of simultaneous measurements of motion and calcium flux. Not requires special equipment.	Not detecting the direct contractile force.	Huebsch, 2015^45^
Cantilevers	3D cardiac tissue	Contractile force	Device integrated with a poly-PDMS encapsulated crack sensor to measure the contractile force.	Detecting the direct contractile force. Long-term evaluation	Low throughput. Requires large equipment for detection of contractile force. Costly. Low sensitivity.	Kim, 2020^46^
Fibrin gel sheets and force transducer	3D cardiac cell sheet	Contractile force	Creates dynamically beating cardiac cell sheet-tissues by attaching the cell sheets on fibrin gel sheets and measures the contractile force by force transducer.	Detecting the direct contractile force. Long-term evaluation.	Low throughput. Requires large equipment for detection of contractile force. Costly. Low sensitivity.	Sasaki, 2018^47^ Gao, 2019^48^
3D heart-on-a-chip microdevice	3D cardiac tissue	Drug response	A pneumatically actuated heart-on-a-chip platform generates uniaxial cyclic strain that drives iPSC-CMs toward maturation.	The ability to reproduce the physiological mechanical environment.	There is no mechanism for assessing function, such as pulsatile force directly.	Marsano, 2016^49^
Heart-on-a-chip micro device	3D cardiac cell sheet	Pulsating force	Bioassay system based on MEMS. Quantify the actual pulsating force of the cell sheet by converting it into the amount of displacement of the beads.	Detecting the direct pulsating force. High sensitivity.	Low throughput. Not suitable for long-term evaluation.	Abulaiti, 2020^50^

Abbreviations: PSC-CMs, pluripotent stem cell-derived cardiomyocytes; 2(3)D, two(three) dimensional; PDMS, dimethylpolysiloxane; MEMS, micro electro mechanical systems.

## Advantage of Human PSC-Derived 3D Cardiac Tissues

Various forms of 3D cardiac tissue derived from pluripotent stem cells have been reported to demonstrate higher structural and functional reproduction of the native cardiac environment than single hPSC-CMs or 2D cultures (Fig. 1).^35,38,64–68^ Three-dimensional cardiac tissues, including fibrin-based engineered heart tissue (EHT), as reported by several groups,^69,70^ can be created using special 3D scaffold-based cultures^38,66,71^ or by mixing non-CMs fractions with cardiac endothelial cells, vascular stromal cells, and cardiac fibroblasts in a ratio that reproduces adult cardiac tissues.^68,72^ These 3D cardiac tissues demonstrate a certain degree of maturation in terms of structure, electron transport chain, and expression of various metabolic pathways, and can partially reproduce the effects of cells other than CMs such as the paracrine effect. Although 3D cardiac tissues are not yet ideal adult heart cell substitutes, they are more effective than 2D-cultured CMs in simulating the responsiveness of various human cardiac tissues.

## Systems to Evaluate the Function of 3D Human PSC-Derived Cardiac Tissues

Previously, we discussed the development of more mature engineered cardiac tissues. Moreover, there is an increasing focus on creating systems to evaluate pulsatile forces using pluripotent stem cell-derived 3D heart tissues (Table 1). The contractile force of cardiac muscle cells has been commonly evaluated using image analyses, such as MUSCLEMOTION.^44^ In addition, computer motion tracking software has been reported to be based on algorithms that generate vector fields that can be used to quantify the spatial distribution of beats in tissue structures^45^ and algorithms that integrate multiple parameters like videos of single beating cells, of microbead displacement during contractions, and of fluorescently labeled myofibrils.^73^ Although these programs are designed to analyze contractile forces easily, these systems do not completely recapitulate the actual pulsatile force of the heart because they are calculated through the temporal changes in serially collected images, which might not fully reflect the physical environment of the heart. Recently, several methods have been reported to evaluate the pulsating force in heart tissues. The measurement system reported by Sasaki et al directly measured contractile forces by placing a cell sheet consisting of hPSC-CMs on a fibrin gel sheet and setting it on a force transducer. They demonstrated that the contractile force of cardiac muscle cells could be detected directly and stably for a long time. The contractile force detected from the hPSC-CMs cell sheet was approximately 3.3 mN/mm^2^.^47,48^ Kim et al reported a system for measuring the contractile force of cardiac muscle cells using a cantilever. The crack in the cantilever opens and closes in response to the contraction of cardiac muscle cells. The magnitude of this opening and closing is correlated with the actual contractile force of the CMs themselves.^46^ Nevertheless, even though these technological advances in the direct measurement of tissue function in 3D cardiac tissues have been achieved, further developments are anticipated regarding the establishment of a high-throughput cardiotoxicity assessment system considering the requirements of special and large-scale equipment and operation costs in the aforementioned systems.

**Figure 2.**
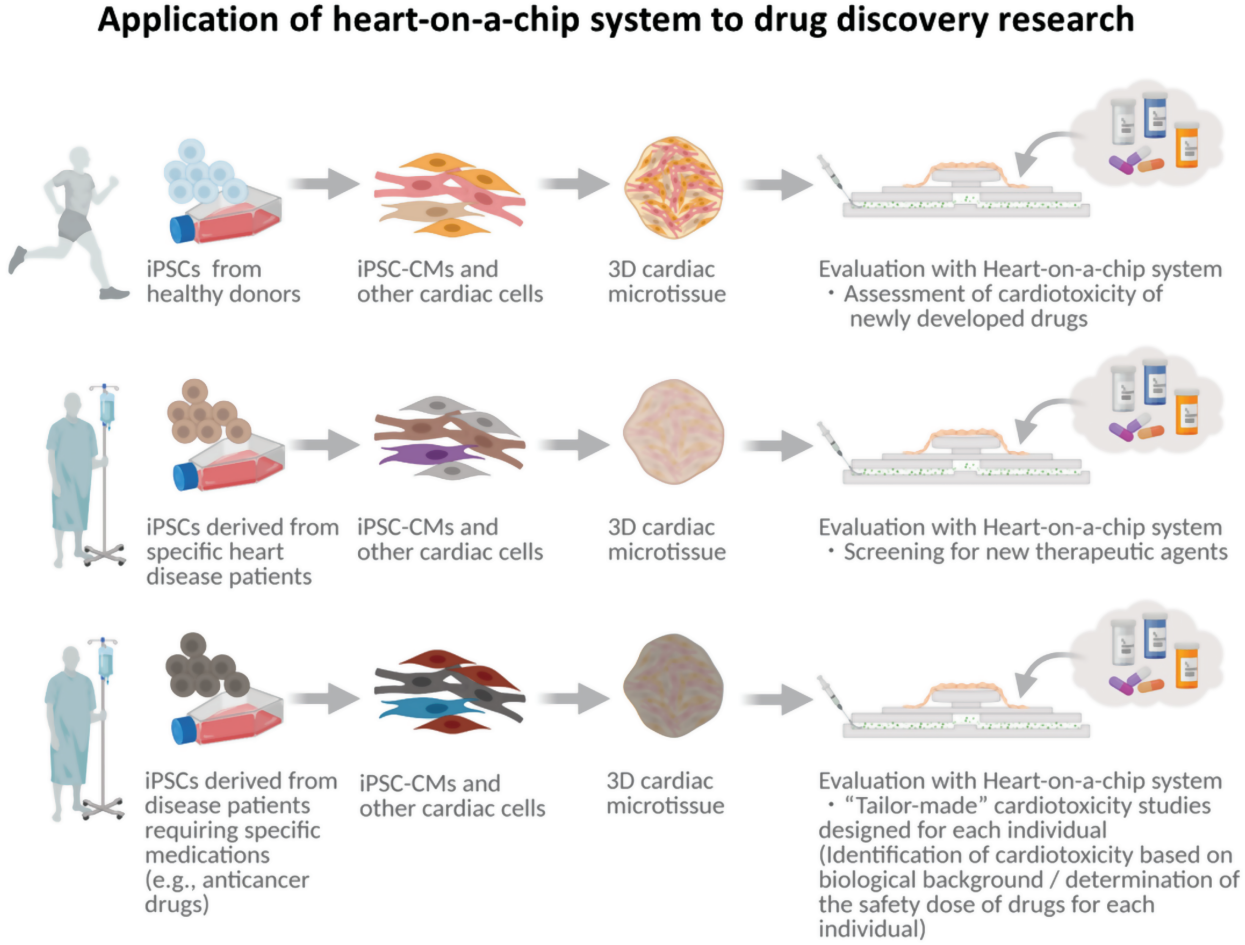
The application of heart-on-a-chip systems in drug discovery research and regenerative medicine. Abbreviations: 3D hPSC-CMs, three-dimensional cardiomyocytes induced from human pluripotent stem cells; 2D hPSC-CMs, 2-dimensional cardiomyocytes induced from human pluripotent stem cells.

As an alternative approach, organ-on-a-chip technology is a new concept that utilizes micro electro mechanical systems (MEMS) to reproduce organ functions using polymeric organosilicon compounds such as polydimethylsiloxane (PDMS), and is applied for the construction of various bioassay systems.^74–76^ The heart-on-a-chip, an organ-on-a-chip that mimics the function of the heart, has also been developed.^49^ Using MEMS-based organ-on-a-chip technology with microfluidic systems, heart-on-a-chip could reproduce cardiac physiological pump function^50,77,78^ and are expected to be widely used in medical research (Fig. 2). The heart-on-a-chip systems might serve as a bioassay system that can predict human cardiotoxicity during the development of new drugs. The ability to accurately assess and screen human cardiotoxicity at the early stages of drug development, such as in basic research or in pre-clinical studies, poses a significant advantage since it is costly to withdraw drugs after they have been released into the market. Furthermore, heart-on-a-chip can be generated from iPSCs established from somatic cells of patients with specific hereditary diseases, such as familial dilated cardiomyopathy, to develop new therapeutic drugs through screening tests using chemical libraries in which functional tissue recovery by the administration of candidate chemicals can be evaluated. Heart-on-a-chip systems could also potentially be used to establish “tailor-made” cardiac toxicity studies. For example, systems can be prepared with iPSCs-derived from patients requiring specific medications, such as anticancer drugs. These are then used to identify acceptable drug dosages according to the individuals’ biological backgrounds. Furthermore, the capability of the heart-on-a-chip system to assess physiological parameters of the heart would further contribute to the evaluation of the maturation level of hPSC-CMs and 3D hPSC-CMs derived tissues. However, in order to sufficiently reproduce the function of the human adult heart using the heart-on-a-chip systems, further progress in the development and standardization of appropriate culture systems of 3D hPSC-derived heart tissues to realize optimized structure and cellular components recapitulating those in the human adult heart is required.

## Closing remarks

In the present review, we introduced the challenges of PSC-based drug discovery research, especially in the evaluation of cardiotoxicity, and the latest devices that can be used to address these challenges. Bioassay systems such as heart-on-a-chip which can evaluate physical parameters of heart tissue might contribute to a wide range of research fields including regenerative medicine and drug discovery.

## Data Availability

No new data were generated or analyzed in support of this research.

## References

[1] Virani SS , Alonso A, Benjamin EJ, et al. Heart disease and stroke statistics—2020 update: a report from the American Heart Association. Circulation. 2020; 141(9):e139-e596 31992061 10.1161/CIR.0000000000000757

[2] Mangini S , Alves BR, Silvestre OM, et al. Heart transplantation: review. Einstein (Sao Paulo). 2015;13:310-318. 10.1590/S1679-45082015RW3154. 26154552 PMC4943829

[3] Packer M , Bristow MR, Cohn JN, et al. The effect of carvedilol on morbidity and mortality in patients with chronic heart failure. N Engl J Med. 1996;334:1349-1355. 10.1056/NEJM199605233342101. 8614419

[4] Lechat P. The Cardiac Insufficiency Bisoprolol Study II (CIBIS-II): a randomised trial. Lancet. 1999;353:9-131. 10023943

[5] Lian X , Hsiao C, Wilson G, et al. Robust cardiomyocyte differentiation from human pluripotent stem cells via temporal modulation of canonical Wnt signaling. Proc Natl Acad Sci USA. 2012;109:E1848-E1857. 22645348 10.1073/pnas.1200250109 PMC3390875

[6] van Laake LW , Passier R, Monshouwer-Kloots J, et al. Human embryonic stem cell-derived cardiomyocytes survive and mature in the mouse heart and transiently improve function after myocardial infarction. Stem Cell Res. 2007;1:9-24. 19383383 10.1016/j.scr.2007.06.001

[7] Chen VC , Ye J, Shukla P, et al. Development of a scalable suspension culture for cardiac differentiation from human pluripotent stem cells. Stem Cell Res. 2015;15:365-375. 10.1016/j.scr.2015.08.002. 26318718 PMC4600677

[8] Yamashita JK , Takano M, Hiraoka-Kanie M, et al. Prospective identification of cardiac progenitors by a novel single cell-based cardiomyocyte induction. FASEB J. 2005;19:1534-1536. 10.1096/fj.04-3540fje. 16033809

[9] Orlic D , Kajstura J, Chimenti S, et al. Bone marrow cells regenerate infarcted myocardium. Nature. 2001;410:701-705. 10.1038/35070587. 11287958

[10] Meyer GP , Wollert KC, Lotz J, et al. Intracoronary bone marrow cell transfer after myocardial infarction: 5-year follow-up from the randomized-controlled BOOST trial. Eur Heart J. 2009;30:2978-2984. 10.1093/eurheartj/ehp374. 19773226

[11] Perin EC , Willerson JT, Pepine CJ, et al. Effect of transendocardial delivery of autologous bone marrow mononuclear cells on functional capacity, left ventricular function, and perfusion in chronic ischemic heart failure: the FOCUS-CCTRN trial. JAMA. 2012;307:1717-1726. 10.1001/jama.2012.418. 22447880 PMC3600947

[12] Hare JM , Fishman JE, Gerstenblith G, et al. Comparison of allogeneic vs autologous bone marrow-derived mesenchymal stem cells delivered by transendocardial injection in patients with ischemic cardiomyopathy. J Autism Dev Disord. 2017;47:549-562.27853923

[13] Menasché P. Cell therapy with human ESC-derived cardiac cells: clinical perspectives. Front Bioeng Biotechnol. 2020;8:601560. 33195177 10.3389/fbioe.2020.601560 PMC7649799

[14] Idänpään-Heikkilä JE. Pharmacogenetics towards improving treatment with medicines. CIOMS. 2005:4–224.

[15] Barth E , Stammler G, Speiser B, et al. Ultrastructural quantitation of mitochondria and myofilaments in cardiac muscle from 10 different animal species including man. J Mol Cell Cardiol. 1992;681:669-681. 10.1016/0022-2828(92)93381-s 1404407

[16] Onakpoya IJ , Heneghan CJ, Aronson JK. Post-marketing withdrawal of 462 medicinal products because of adverse drug reactions: a systematic review of the world literature. BMC Med. 2016;14:1-11. 26843061 10.1186/s12916-016-0553-2 PMC4740994

[17] Álamo JC del , Lemons D, Serrano R, et al. High throughput physiological screening of iPSC-derived cardiomyocytes for drug development. Biochim Biophys Acta. 2016;1863:1717-1727. 26952934 10.1016/j.bbamcr.2016.03.003 PMC4885786

[18] Ando H , Yoshinaga T, Yamamoto W, et al. A new paradigm for drug-induced torsadogenic risk assessment using human iPS cell-derived cardiomyocytes. J Pharmacol Toxicol Methods. 2017;84:111-127. 10.1016/j.vascn.2016.12.003. 27956204

[19] Blinova K , Dang Q, Millard D, et al. International multisite study of human-induced pluripotent stem cell-derived cardiomyocytes for drug proarrhythmic potential assessment. Cell Rep. 2018;24:3582-3592. 10.1016/j.celrep.2018.08.079. 30257217 PMC6226030

[20] Cerignoli F , Charlot D, Whittaker R, et al. High throughput measurement of Ca2+ dynamics for drug risk assessment in human stem cell-derived cardiomyocytes by kinetic image cytometry. J Pharmacol Toxicol Methods. 2012;66:246-256. 10.1016/j.vascn.2012.08.167. 22926323 PMC3667588

[21] Lu HR , Whittaker R, Price JH, et al. High throughput measurement of Ca^++^ dynamics in human stem cell-derived cardiomyocytes by kinetic image cytometery: a cardiac risk assessment characterization using a large panel of cardioactive and inactive compounds. Toxicol Sci. 2015;148:503-516. 10.1093/toxsci/kfv201. 26358003

[22] Wright PT , Tsui SF, Francis AJ, et al. Approaches to high-throughput analysis of cardiomyocyte contractility. Front Physiol. 2020;11:1-19. 32733259 10.3389/fphys.2020.00612 PMC7362994

[23] Kattman SJ , Witty AD, Gagliardi M, et al. Stage-specific optimization of activin/nodal and BMP signaling promotes cardiac differentiation of mouse and human pluripotent stem cell lines. Cell Stem Cell. 2011;8:228-240. 10.1016/j.stem.2010.12.008. 21295278

[24] Yang L , Soonpaa MH, Adler ED, et al. Human cardiovascular progenitor cells develop from a KDR+ embryonic-stem-cell-derived population. Nature. 2008;453:524-528. 10.1038/nature06894. 18432194

[25] Laflamme MA , Chen KY, Naumova AV, et al. Cardiomyocytes derived from human embryonic stem cells in pro-survival factors enhance function of infarcted rat hearts. Nat Biotechnol. 2007;25:1015-1024. 17721512 10.1038/nbt1327

[26] Willems E , Spiering S, Davidovics H, et al. Small-molecule inhibitors of the Wnt pathway potently promote cardiomyocytes from human embryonic stem cell-derived mesoderm. Circ Res. 2011;109:360-364. 10.1161/CIRCRESAHA.111.249540. 21737789 PMC3327303

[27] Burridge PW , Anderson D, Priddle H, et al. Improved human embryonic stem cell embryoid body homogeneity and cardiomyocyte differentiation from a novel V-96 plate aggregation system highlights interline variability. Stem Cells. 2007;25:929-938. 10.1634/stemcells.2006-0598. 17185609

[28] Hudson J , Titmarsh D, Hidalgo A, et al. Primitive cardiac cells from human embryonic stem cells. Stem Cells Dev. 2011;21:1513-1523. 21933026 10.1089/scd.2011.0254

[29] Uosaki H , Fukushima H, Takeuchi A, et al. Efficient and scalable purification of cardiomyocytes from human embryonic and induced pluripotent stem cells by VCAM1 surface expression. PLoS One. 2011;6:e23657. 10.1371/journal.pone.0023657. 21876760 PMC3158088

[30] Burridge PW , Keller G, Gold JD, et al. Production of de novo cardiomyocytes: human pluripotent stem cell differentiation and direct reprogramming. Cell Stem Cell. 2012;10:16-28. 10.1016/j.stem.2011.12.013. 22226352 PMC3255078

[31] Cavero I , Crumb W. ICH S7B draft guideline on the non-clinical strategy for testing delayed cardiac repolarisation risk of drugs: a critical analysis. Expert Opin Drug Saf. 2005;4:509-530. 10.1517/14740338.4.3.509. 15934857

[32] Redfern WS , Carlsson L, Davis AS, et al. Relationships between preclinical cardiac electrophysiology, clinical QT interval prolongation and torsade de pointes for a broad range of drugs: evidence for a provisional safety margin in drug development. Cardiovasc Res. 2003;58:32-45. 10.1016/s0008-6363(02)00846-5. 12667944

[33] Liang P , Lan F, Lee AS, et al. Drug screening using a library of human induced pluripotent stem cell-derived cardiomyocytes reveals disease-specific patterns of cardiotoxicity. Circulation. 2013;127:1677-1691. 10.1161/CIRCULATIONAHA.113.001883. 23519760 PMC3870148

[34] Yamazaki D , Kitaguchi T, Ishimura M, et al. Proarrhythmia risk prediction using human induced pluripotent stem cell-derived cardiomyocytes. J Pharmacol Sci. 2018;136:249-256. 10.1016/j.jphs.2018.02.005. 29555184

[35] Sacchetto C , Vitiello L, de Windt LJ, et al. Modeling cardiovascular diseases with hipsc-derived cardiomyocytes in 2d and 3d cultures. Int J Mol Sci. 2020;21(9):3404. 10.3390/ijms21093404. PMC7246991 32403456

[36] Karbassi E , Fenix A, Marchiano S, et al. Implications for regenerative medicine. Nat Rev Cardiol. 2020;17:341-359. 10.1038/s41569-019-0331-x. 32015528 PMC7239749

[37] Protze SI , Lee JH, Keller GM. Human pluripotent stem cell-derived cardiovascular cells: from developmental biology to therapeutic applications. Cell Stem Cell. 2019;25:311-327. 10.1016/j.stem.2019.07.010. 31491395

[38] Ronaldson-Bouchard K , Ma SP, Yeager K, et al. Advanced maturation of human cardiac tissue grown from pluripotent stem cells. Nature. 2018;556:239-243. 10.1038/s41586-018-0016-3. 29618819 PMC5895513

[39] Mills RJ , Titmarsh DM, Koenig X, et al. Functional screening in human cardiac organoids reveals a metabolic mechanism for cardiomyocyte cell cycle arrest. Proc Natl Acad Sci USA. 2017;114:E8372-E8381. 10.1073/pnas.1707316114. 28916735 PMC5635889

[40] Hu D , Linders A, Yamak A, et al. Metabolic maturation of human pluripotent stem cellderived cardiomyocytes by inhibition of HIF1α and LDHA. Circ Res. 2018;123:1066-1079. 10.1161/CIRCRESAHA.118.313249. 30355156 PMC6208155

[41] Ribeiro AJS , Ang YS, Fu JD, et al. Contractility of single cardiomyocytes differentiated from pluripotent stem cells depends on physiological shape and substrate stiffness. Proc Natl Acad Sci USA. 2015;112:12705-12710. 10.1073/pnas.1508073112. 26417073 PMC4611612

[42] Sirenko O , Crittenden C, Callamaras N, et al. Multiparameter in vitro assessment of compound effects on cardiomyocyte physiology using iPSC cells. J Biomol Screen. 2013;18:39-53. 10.1177/1087057112457590. 22972846

[43] Lewis KJ , Silvester NC, Barberini-Jammaers S, et al. A new system for profiling drug-induced calcium signal perturbation in human embryonic stem cell-derived cardiomyocytes. J Biomol Screen. 2015;20:330-340. 10.1177/1087057114557232. 25367900 PMC4361473

[44] Sala L , Van Meer BJ, Tertoolen LGJ, et al. Musclemotion: a versatile open software tool to quantify cardiomyocyte and cardiac muscle contraction in vitro and in vivo. Circ Res. 2018;122:e5-e16. 29282212 10.1161/CIRCRESAHA.117.312067 PMC5805275

[45] Huebsch N , Loskill P, Mandegar MA, et al. Automated video-based analysis of contractility and calcium flux in human-induced pluripotent stem cell-derived cardiomyocytes cultured over different spatial scales. Tissue Eng – Part C Methods. 2015;21:467-479. 25333967 10.1089/ten.tec.2014.0283 PMC4410286

[46] Kim DS , Choi YW, Shanmugasundaram A, et al. Highly durable crack sensor integrated with silicone rubber cantilever for measuring cardiac contractility. Nat Commun. 2020;11:1-13. 31988308 10.1038/s41467-019-14019-y PMC6985253

[47] Sasaki D , Matsuura K, Seta H, et al. Contractile force measurement of human induced pluripotent stem cell-derived cardiac cell sheet-tissue. PLoS One. 2018;13:e01980261-e01980221. 10.1371/journal.pone.0198026. PMC5965888 29791489

[48] Gao B , Matsuura K, Shimizu T. Recent progress in induced pluripotent stem cell-derived cardiac cell sheets for tissue engineering. Biosci Trends. 2019;13:292-298. 10.5582/bst.2019.01227. 31527326

[49] Marsano A , Conficconi C, Lemme M, et al. Beating heart on a chip: a novel microfluidic platform to generate functional 3D cardiac microtissues. Lab Chip. 2016;16:599-610. 10.1039/c5lc01356a. 26758922

[50] Abulaiti M , Yalikun Y, Murata K, et al. Establishment of a heart-on-a-chip microdevice based on human iPS cells for the evaluation of human heart tissue function. Sci Rep. 2020;10:1-12. 33154509 10.1038/s41598-020-76062-w PMC7645446

[51] Clements M , Millar V, Williams AS, et al. Bridging functional and structural cardiotoxicity assays using human embryonic stem cell-derived cardiomyocytes for a more comprehensive risk assessment. Toxicol Sci. 2015;148:241-260. 10.1093/toxsci/kfv180. 26259608

[52] Caspi O , Itzhaki I, Kehat I, et al. In vitro electrophysiological drug testing using human embryonic stem cell derived cardiomyocytes. Stem Cells Dev. 2008;18:161-172. 10.1089/scd.2007.0280 18510453

[53] Peng S , Lacerda AE, Kirsch GE, et al. The action potential and comparative pharmacology of stem cell-derived human cardiomyocytes. J Pharmacol Toxicol Methods. 2010;61:277-286. 10.1016/j.vascn.2010.01.014. 20153443

[54] Edwards SL , Zlochiver V, Conrad DB, et al. A multiwell cardiac μGMEA platform for action potential recordings from human iPSC-derived cardiomyocyte constructs. Stem Cell Rep. 2018;11:522-536. 10.1016/j.stemcr.2018.06.016. PMC6092761 30033088

[55] Guo L , Coyle L, Abrams RMC, et al. Refining the human iPSC-cardiomyocyte arrhythmic risk assessment model. Toxicol Sci. 2013;136:581-594. 10.1093/toxsci/kft205. 24052561

[56] Pointon A , Abi-gerges N, Cross MJ, et al. Phenotypic profiling of structural cardiotoxins in vitro reveals dependency on multiple mechanisms of toxicity. Toxicol Sci. 2013;132:317-326. 10.1093/toxsci/kft005. 23315586

[57] Wahlquist C , Jeong D, Rojas-Muñoz A, et al. Inhibition of miR-25 improves cardiac contractility in the failing heart. Nature. 2014;508:531-535. 10.1038/nature13073. 24670661 PMC4131725

[58] Agarwala A , Gossa JA, Choa A, et al. Microfluidic heart on a chip for higher throughput pharmacological studies. Lab Chip. 2013;13:3599-3608. 23807141 10.1039/c3lc50350j PMC3786400

[59] Rana P , Anson B, Engle S, et al. Characterization of human-induced pluripotent stem cell-derived cardiomyocytes: bioenergetics and utilization in safety screening. Toxicol Sci. 2012;130:117-131. 10.1093/toxsci/kfs233. 22843568

[60] Gupta MK , Illich DJ, Gaarz A, et al. Global transcriptional profiles of beating clusters derived from human induced pluripotent stem cells and embryonic stem cells are highly similar. BMC Dev Biol. 2010;10. 10.1186/1471-213X-10-98 PMC2946283 20843318

[61] Xiu QX , Set YS, Sun W, et al. Global expression profile of highly enriched cardiomyocytes derived from human embryonic stem cells. Stem Cells. 2009;27:2163-2174. 19658189 10.1002/stem.166

[62] Van Den Berg CW , Okawa S, Chuva De Sousa Lopes SM, et al. Transcriptome of human foetal heart compared with cardiomyocytes from pluripotent stem cells. Development. 2015;142:3231-3238. 26209647 10.1242/dev.123810

[63] Yang X , Pabon L, Murry CE. Engineering adolescence: maturation of human pluripotent stem cell-derived cardiomyocytes. Circ Res. 2014;114:511-523. 10.1161/CIRCRESAHA.114.300558. 24481842 PMC3955370

[64] Thomas D , Cunningham NJ, Shenoy S, et al. Human iPSCs in Cardiovascular research: current approaches in cardiac differentiation, maturation strategies, and scalable production. Cardiovasc Res. 2021;1:17. 10.1093/cvr/cvab115 PMC8932155 33757124

[65] Simon LTR , Masters KS. Disease-inspired tissue engineering: investigation of cardiovascular pathologies. ACS Biomater Sci Eng. 2020;6:2518-2532. 32974421 10.1021/acsbiomaterials.9b01067 PMC7508300

[66] Nunes SS , Miklas JW, Liu J, et al. Biowire: a new platform for maturation of human pluripotent stem cell derived cardiomyocytes. Nat Methods. 2014;10:781-787. 10.1038/nmeth.2524 PMC4071061 23793239

[67] Giacomelli E , Bellin M, Sala L, et al. Three-dimensional cardiac microtissues composed of cardiomyocytes and endothelial cells co-differentiated from human pluripotent stem cells. Development. 2017;144:1008-1017. 28279973 10.1242/dev.143438 PMC5358113

[68] Giacomelli E , Meraviglia V, Campostrini G, et al. Human-iPSC-derived cardiac stromal cells enhance maturation in 3D cardiac microtissues and reveal non-cardiomyocyte contributions to heart disease. Cell Stem Cell. 2020;26:862-879.e11. 10.1016/j.stem.2020.05.004. 32459996 PMC7284308

[69] Eschenhagen T , Fink C, Remmers U, et al. Three-dimensional reconstitution of embryonic cardiomyocytes in a collagen matrix: a new heart muscle model system. FASEB J. 1997;11:683-694. 10.1096/fasebj.11.8.9240969. 9240969

[70] Zimmermann WH , Melnychenko I, Eschenhagen T. Engineered heart tissue for regeneration of diseased hearts. Biomaterials. 2004;25:1639-1647. 10.1016/s0142-9612(03)00521-0. 14697865

[71] Lemoine MD , Mannhardt I, Breckwoldt K, et al. Human iPSC-derived cardiomyocytes cultured in 3D engineered heart tissue show physiological upstroke velocity and sodium current density. Sci Rep. 2017;7:1-11. 28710467 10.1038/s41598-017-05600-w PMC5511281

[72] Masumoto H , Nakane T, Tinney JP, et al. The myocardial regenerative potential of three-dimensional engineered cardiac tissues composed of multiple human iPS cell-derived cardiovascular cell lineages. Sci Rep. 2016;6:1-10. 27435115 10.1038/srep29933 PMC4951692

[73] Ribeiro AJS , Schwab O, Mandegar MA, et al. Multi-imaging method to assay the contractile mechanical output of micropatterned human iPSC-derived cardiac myocytes. Circ Res. 2017;120(10):1572-1583. 10.1161/CIRCRESAHA.116.310363. 28400398 PMC5491345

[74] Tanaka Y , Morishima K, Shimizu T, et al. An actuated pump on-chip powered by cultured cardiomyocytes. Lab Chip. 2006;6:362-368. 10.1039/b515149j. 16511618

[75] Huh D , Matthews BD, Mammoto A, et al. Reconstituting organ-level lung. Science (80-). 2010:1662–1668. 20576885 10.1126/science.1188302 PMC8335790

[76] Zhao Y , Rafatian N, Wang EY, et al. Engineering microenvironment for human cardiac tissue assembly in heart-on-a-chip platform. Matrix Biol. 2020;85–86:189-204. 10.1016/j.matbio.2019.04.001 PMC6788963 30981898

[77] Tanaka Y , Fujita H. Fluid driving system for a micropump by differentiating iPS cells into cardiomyocytes on a tent-like structure. Sens Actuators, B Chem. 2015;210:267-272.

[78] Tanaka Y , Yanagisawa Y, Kitamori T. Fluid actuation for a bio-micropump powered by previously frozen cardiomyocytes directly seeded on a diagonally stretched thin membrane. Sens Actuators, B Chem. 2011;156:494-498.

